# Novel missense *ACAN* gene variants linked to familial osteochondritis dissecans cluster in the C-terminal globular domain of aggrecan

**DOI:** 10.1038/s41598-022-09211-y

**Published:** 2022-03-25

**Authors:** Eva-Lena Stattin, Karin Lindblom, André Struglics, Patrik Önnerfjord, Jack Goldblatt, Abhijit Dixit, Ajoy Sarkar, Tabitha Randell, Mohnish Suri, Cathleen Raggio, Jessica Davis, Erin Carter, Anders Aspberg

**Affiliations:** 1 Department of Immunology, Genetics and Pathology, Uppsala University, Uppsala, Sweden; 2 Rheumatology and Molecular Skeletal Biology, Department of Clinical Sciences Lund, Lund University, BMC-C12, 22184 Lund, Sweden; 3 Orthopaedics, Department of Clinical Sciences Lund, Lund University, Lund, Sweden; 4 Genetic Services & Familial Cancer Program of Western Australia, King Edward Memorial Hospital for Women, Perth, WA Australia; 5 Department of Clinical Genetics, Nottingham University Hospitals NHS Trust, Nottingham, UK; 6 Department of Paediatric Endocrinology, Nottingham University Hospitals NHS Trust, Nottingham, UK; 7 Kathryn O. and Alan C. Greenberg Center for Skeletal Dysplasias, Hospital for Special Surgery, New York, NY USA

**Keywords:** Cartilage, Disease genetics, Genetic predisposition to disease, Musculoskeletal abnormalities

## Abstract

The cartilage aggrecan proteoglycan is crucial for both skeletal growth and articular cartilage function. A number of aggrecan (*ACAN)* gene variants have been linked to skeletal disorders, ranging from short stature to severe chondrodyplasias. Osteochondritis dissecans is a disorder where articular cartilage and subchondral bone fragments come loose from the articular surface. We previously reported a missense *ACAN* variant linked to familial osteochondritis dissecans, with short stature and early onset osteoarthritis, and now describe three novel *ACAN* gene variants from additional families with this disorder. Like the previously described variant, these are autosomal dominant missense variants, resulting in single amino acid residue substitutions in the C-type lectin repeat of the aggrecan G3 domain. Functional studies showed that neither recombinant variant proteins, nor full-length variant aggrecan proteoglycan from heterozygous patient cartilage, were secreted to the same level as wild-type aggrecan. The variant proteins also showed decreased binding to known cartilage extracellular matrix ligands. Mapping these and other *ACAN* variants linked to hereditary skeletal disorders showed a clustering of osteochondritis dissecans-linked variants to the G3 domain. Taken together, this supports a link between missense *ACAN* variants affecting the aggrecan G3 domain and hereditary osteochondritis dissecans.

## Introduction

The large proteoglycan aggrecan is vital for skeletal development and cartilage function, creating a swelling pressure in the tissue that provides resistance to compression and load dissipation over the joint surface^1^. Aggrecan contains an extended central region carrying keratan sulfate and chondroitin sulfate chains. This region is flanked by globular protein domains, the N-terminal G1 and G2 domains and the C-terminal G3 domain, mediating interactions with other components of the extracellular matrix^2^.

A complete lack of aggrecan causes severe chondrodysplasia and is incompatible with postnatal life, as observed in mice^3^, cattle^4^ and chick^5^. In humans, several aggrecan (*ACAN*) gene variants have been linked to hereditary skeletal disorders. Some of these variants are linked to the skeletal dysplasias spondyloepiphyseal dysplasia Kimberley type (SEDK)^6^, and spondyloepimetaphyseal dysplasia aggrecan type (SEMD)^7^. In recent years multiple additional *ACAN* gene variants have been identified, many of which are variants of unknown significance (VUS) or linked to milder phenotypes such as idiopathic short stature (Supplemental Table 1)^6,8–34^. While heredity has not been confirmed for some of these variants, most show clear segregation with disease.

Most *ACAN* variants result in truncation or haploinsufficiency due to premature stop codons, or in nonsense mediated decay of the aggrecan mRNA as shown for the bovine Dexter Bulldog *ACAN* variant^4^. Less is known concerning disease mechanisms for *ACAN* missense variants. These have predominantly been found in the globular domains of aggrecan and are linked to idiopathic short stature. Two of the *ACAN* missense variants have been studied in more detail. The SEMD missense variant p.D2419N (originally referred to as p.D2267N, see Table 1 for updated variant nomenclature) affects the C-type lectin of the G3 globular domain of aggrecan^7^. This recessively inherited variant results in the formation of a novel N-linked glycosylation site, potentially blocking interactions of the C-type lectin. Another missense *ACAN* variant was linked to familial osteochondritis dissecans (fOCD) with short stature and early onset osteoarthritis^8–10^. This variant results in a single amino acid substitution p.V2455M (originally referred to as p.V2303M, see Table 1) in the C-type lectin repeat of the C-terminal G3 globular domain of the protein, affecting intermolecular interactions with other cartilage extracellular matrix components^9^.

**Table 1:** Aggrecan G3 missense variant nomenclature.

Disease	OMIM #	cDNA	Protein	Previously published as (Refseq ID)	Reference
fOCD^a^/SSOAOD^b^		c.7156T>A	p.C2386S	–	Family 1, this study
SSOAOD	165800	c.7178T>C	p.L2393P	c.7064T>C, p.L2355P (NM_013227.3)	^8^
fOCD/SSOAOD		c.7250T>C	p.L2417P	–	Family 2, this study
SEMD-ACAN^c^	612813	c.7255G>A	p.D2419N	c.6799G>A, p.D2267N (NM_013227.2)	^7^
fOCD/SSOAOD		c.7358A>T	p.D2453V	–	Family 3, this study
fOCD/SSOAOD	165800	c.7363G>A	p.V2455M	c.6907G>A, p.V2303M (NM_013227.2)	^9^

The *ACAN* variant cDNA and protein numbering used in the present study is based on RefSeq NM_001369268.1 (PRI 14-OCT-2019).

^a^Familial osteochondritis dissecans with short stature and early onset osteoarthritis. Autosomal dominant inheritance.

^b^Short stature and advanced bone age, with or without early-onset osteoarthritis and/or osteochondritis dissecans. Autosomal dominant inheritance.

^c^Spondyloepimetaphyseal dysplasia, aggrecan type. Autosomal recessive inheritance.

Osteochondritis dissecans (OCD) is a joint disease with partial or total focal detachment of articular cartilage and underlying subchondral bone fragments. The majority of OCD cases are sporadic with a knee OCD prevalence of 15–29 per 100,000 reported in Sweden^11^. The etiology of OCD remains unclear, but is likely multifactorial. Several causes have been suggested, including trauma or microfracture, ischemia, and genetic predisposition^12^. A rare hereditary form, fOCD, presents as multiple osteochondral lesions in knees and/or hips and/or elbows, disproportionate short stature, and early osteoarthritis^10^. We previously identified an association of the p.V2455M *ACAN* variant with fOCD^9^. Since then, other *ACAN* variants linked to fOCD have been reported^8,13,14^. Additional *ACAN* variants have also been described where only the probands present with OCD^8,15–20^. Interestingly, among these, the *ACAN* missense variant p.L2393P (originally referred to as p.L2355P, see Table 1) is located in the G3 domain of aggrecan, like the p.V2455M fOCD variant, and result in short stature with early onset osteoarthritis and with OCD observed in the proband, although heredity for the latter remains unclear^8,17^. In view of this, it is perhaps not surprising that there has been a call for more studies on gene variants linked to fOCD^21^.

In this paper we describe three novel missense *ACAN* gene variants in families with hereditary OCD, short stature and early onset osteoarthritis. These novel variants are missense mutations, affect different amino acid residues in the C-type lectin repeat of aggrecan, and result in decreased secretion and disturbed interactions of the variant protein.

## Results

### Aggrecan G3 variant nomenclature

Earlier studies have based the *ACAN* gene variant position numbering on different reference sequences. Unfortunately, neither of these cover the entire coding sequence of aggrecan. This is due to alternative splicing of the *ACAN* mRNA, where the presence or absence of exons coding for EGF or complement regulatory protein-like repeats (CRP, also known as sushi repeats) in the G3 domain results in different aggrecan isoforms. In this study, we base the variant cDNA and protein nomenclature on the recently added reference sequence for *ACAN* variant 3, which includes the complete G3 domain; comprising EGF1 and EGF2, C-type lectin and CRP/sushi repeats (NM_001369268.1, PRI 14-OCT-2019). Table 1 compares this nomenclature with versions used in previous publications^7–9^.

### Family 1 clinical characteristics

A 19-year-old male was referred for molecular genetic analysis, due to short stature, OCD, and a family history of OCD and premature OA (Table 2). He was 155 cm tall, and he had very short 2nd metatarsal bones. He had experienced multiple arthroscopies to remove osteochondral fragments. His mother was 145 cm tall. Her surgical history includes multiple arthroscopies to remove osteochondral fragments, and unilateral hip and knee replacement. The grandfather was 135 cm tall, and had a history of OCD and premature osteoarthritis.

**Table 2:** Phenotype baseline data of individuals included in study.

	Family 1 (p.C2386S)			Family 2 (p.L2417P)			Family 3 (p.D2453V)		
	Index (III:1)	Mother (II:2)	Grand-father (I:1)	Index (III:1)	Sister (III:2)	Mother (II:2)	Index (III:1)	Brother (III:2)	Father (II:1)
Gender (f/m)	M	F	M	M	F	F	F	M	M
Age (years)	29	73	Deceased	16	23	46	31	28	65
Osteochondritis dissecans	Yes	Yes	Yes	Yes	Yes	Yes	Yes	Yes	Yes
Osteoarthritis	Yes	Yes	Yes	No	No	Yes	Yes	No	Yes
Height (cm)	155	145	135	148	139	143	158	156	156
Weight (kg)	NA	NA	NA	50	43	NA	64	74	74
Head circumference (cm)	NA	NA	NA	58	57	56	57	58	58
Arm span (cm)	NA	NA	NA	152	140	NA	156	160	160
Upper arm, forearm length (cm)	NA	NA	NA	NA	NA	NA	26	NA	NA
Hand length (R/L) (cm)	NA	NA	NA	NA	NA	NA	16.4/17.4	15.7/15.7	15.7
GH treatment	–	–	–	Yes	–	–	–	–	–

*NA* not assessed.

### Family 2 clinical characteristics

**Figure 1:**
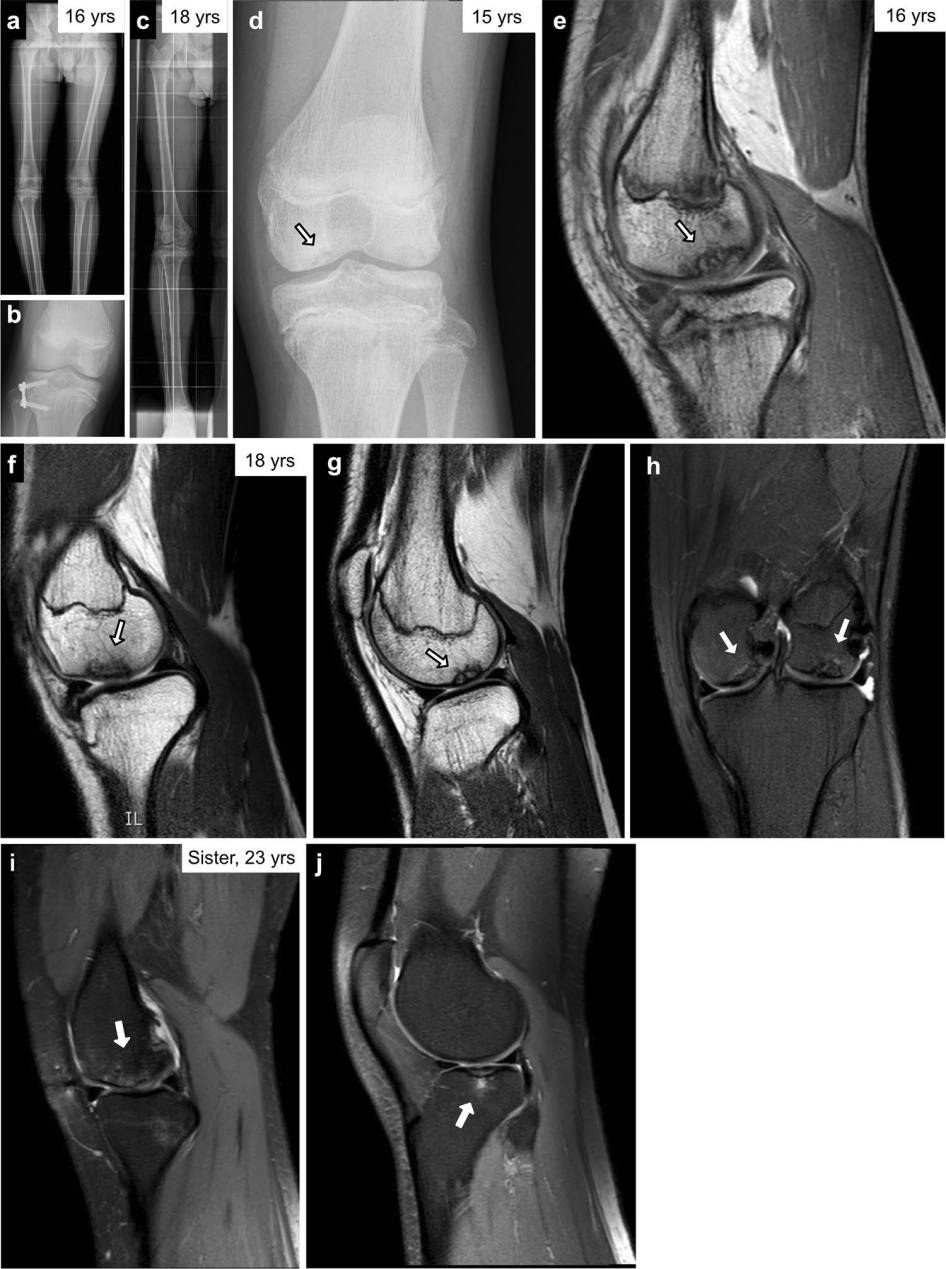
Clinical observations in Family 2. The proband is an 18 years old man with short stature (height 127.5 cm at 13 years old, − 3.5 SDS), normal birth history and development, and a family history of short stature. The proband was treated with GH from the age of 14. At 16 years of age, the proband was still on GH treatment and presented with bilateral knee pain and right-sided genu varum (**a**) that was corrected surgically (**b**,**c**). His knees at 15 (**d**), 16 (**e**) and 18 years of age (**f**–**h**) showed OCD. Both his 23-year-old sister (**i**,**j**) and his 46-year-old mother (143 cm, not shown) have short stature and OCD. Arrows indicate osteochondral lesions.

A 13-year-old male was referred for molecular genetic analysis, due to short stature. He had a normal birth history and development (Table 2). Endocrine evaluation showed partial growth hormone (GH) deficiency, and the boy was treated with GH for 5 years. His height was 127.5 cm (− 3.5 SDS) at 13 years of age. At the age of 16 years, he was 148 cm in stature and still on GH treatment. He had bilateral knee pain with right-sided genu varum that was corrected with proximal tibial lateral 8-plating (Fig. 1a–c). MRI and X-ray of the knee showed OCD (Fig. 1d,e). At the age of 19 he was 155 cm tall, and GH treatment was discontinued. MRI of knees at 18 years confirmed OCD (Fig. 1f–h). Clinical skeletal examination at 16 years showed no abnormalities of the spine, elbows or hands. Family history revealed that his 46-year-old mother, and 23-year-old sister both have short stature (143 cm and 139 cm respectively) and knee OCD initially diagnosed in teenage years (Fig. 1i,j). Both his sister and mother have had multiple right (sister) or bilateral (mother) arthroscopies to remove osteochondral fragments. His mother also has a history of bilateral knee osteoarthritis since her 20s. The 82-year-old maternal grandmother was 139 cm tall, and had bilateral hip replacement for osteoarthritis in her 60s/70s, but there is no confirmed history of OCD. There is also a family history of short stature and knee joint problems, possibly related to OCD in at least two further maternal relatives but their clinical data are not available.

### Family 3 clinical characteristics

**Figure 2:**
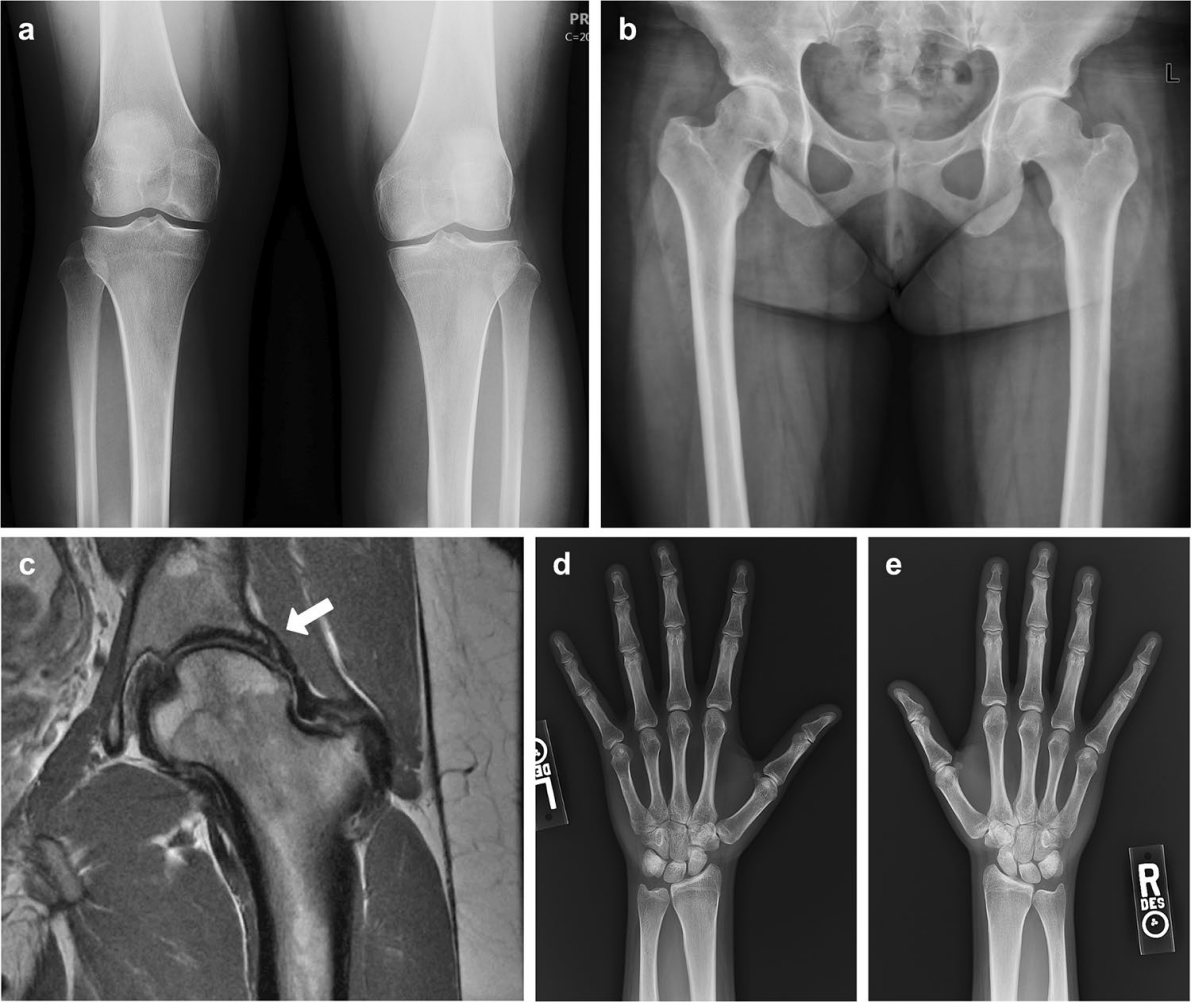
Clinical observations in Family 3. The proband is a now 33-year-old woman, 156 cm tall, with joint pain, early onset osteoarthritis and osteochondral lesions in multiple joints, including knees (**a**) and hips (**b**). She has had hip arthroscopic treatment for femoral-acetabular impingements and labrum tears (**c**, arrow). Hand X-rays show short and broad distal phalanges of the thumbs (**d**,**e**). She has a family history of short stature and joint problems on the paternal side, including OCD.

A 31-year-old woman was referred for molecular genetic analysis, because of joint pain, early-onset degenerative joint disease, and osteochondral lesions in multiple joints (Table 2). Since adolescence she has experienced arthralgia in her hips, knees, ankles, feet, back, shoulders, wrists, and hands. She has mild scoliosis, osteoarthritis, and osteochondral lesions diagnosed on X-ray and MRI studies of her knees and hips (Fig. 2a–c). Surgical history includes left and right hip arthroscopies to treat femoral-acetabular impingement and labrum tears (Fig. 2c). Distal phalanges of the thumbs appear short and broad and she has mild clinodactyly of the 5th fingers (Fig. 2d,e). She has mild myopia (− 0.50) and normal hearing. She was the first child born to non-consanguineous parents of Scotch, Canadian (Nova Scotia), English, and German ancestry, a 26-year-old mother 170 cm tall and a 34-year-old father 155 cm tall. Family history is remarkable in that multiple paternal family members present with short stature and joint problems. The middle child is a brother, currently 28 years old and 155 cm tall, who has a history of knee pain and was said to have a Grade I OCD lesion of the medial femoral condyle on X-ray when he was 11 years old. He has also been told he has issues with his trochleae. Currently he experiences knee pain when running as well as miscellaneous joint cracking. The youngest sibling is a sister, now 25 years old and 150 cm tall; she has dislocated her kneecaps in the past, is said to have trochlear problems, and was diagnosed with a soft tissue sarcoma when she was 18 years old. The father of our proband is currently 65 years old. He has a history of OCD lesions, knee problems, and has had multiple surgeries on his left knee (before the age of 30) and underwent a left total knee replacement in his early 60s. In his youth his parents brought him to the National Institutes of Health (NIH) for evaluation of growth problems. Growth hormone therapy was discussed but not pursued at that time. He was diagnosed with Alzheimer’s disease in his 60s. His mother, i.e. the paternal grandmother of the proband, is now deceased, but was said to be 150 cm tall and had a history of hip and knee problems. Other members of her side of the family were known for being short-statured. She retired early due to joint issues, and eventually underwent hip and knee replacement surgeries. Her husband, the paternal grandfather of our proband, 188 cm tall, is still living at 93 years old. That couple had two other children, a son and a daughter who are said to be well and are said to be 188 cm and 170 cm tall, respectively, and have no known joint problems.

### Molecular genetic analysis

**Figure 3:**
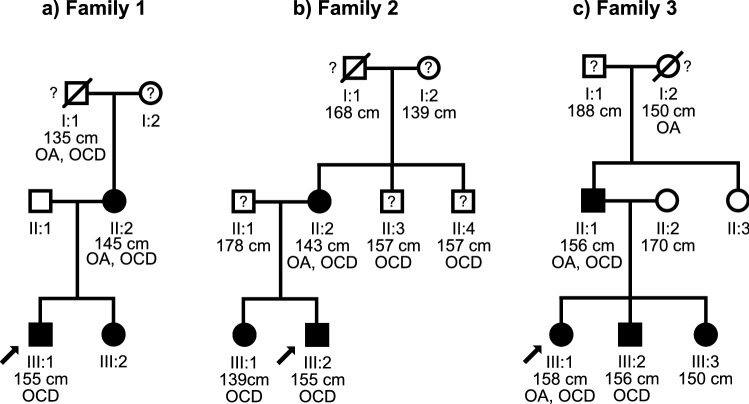
Pedigrees of the studied families. Pedigrees are shown for (**a**) Family 1, from Australia, (**b**) Family 2, from the United Kingdom, and (**c**) Family 3, from New York, USA. The probands are identified by arrows. Filled symbols indicate individuals with heterozygous *ACAN* gene variants verified by DNA sequencing, question marks indicate no sequence information is available for the individual. The height and diagnosed osteoarthritis (OA) or osteochondritis dissecans (OCD) are indicated below symbols.

Targeted sequence analysis of *ACAN* revealed heterozygous likely pathogenic variants in two families. In family 1, we identified a missense variant: c.7156T>A, in exon 15 (p.C2386S) (Supplemental Fig. 1a) in DNA from the proband sent from Australia. The sister and mother of the proband were found to carry the p.C2386S variant (Fig. 3a). The healthy father was not a carrier.

In the second proband referred from UK (family 2), we identified the c.7250T>C, p.L2417P variant, located in exon 16 (Supplemental Fig. 1b). Segregation analysis showed that the p.L2417P variant was present in his affected sister, and mother (Fig. 3b).

In family 3, a third *ACAN* variant in exon 17 was identified by NGS at Fulgent Genetics; c.7358A>T, p.D2453V. This variant was reported to ClinVar NCBI by Fulgent Genetics, as a variant of unclear significance (VUS).Two siblings of the proband as well as the father were found to be heterozygous carriers for the p.D2453V *ACAN* variant by Sanger sequencing (Fig. 3c). The familial variant was not detected in the proband’s mother and a paternal aunt, both of normal stature and neither of whom have any history of orthopedic problems.

### The novel missense ACAN variants affect the C-type lectin repeat of aggrecan

**Figure 4:**
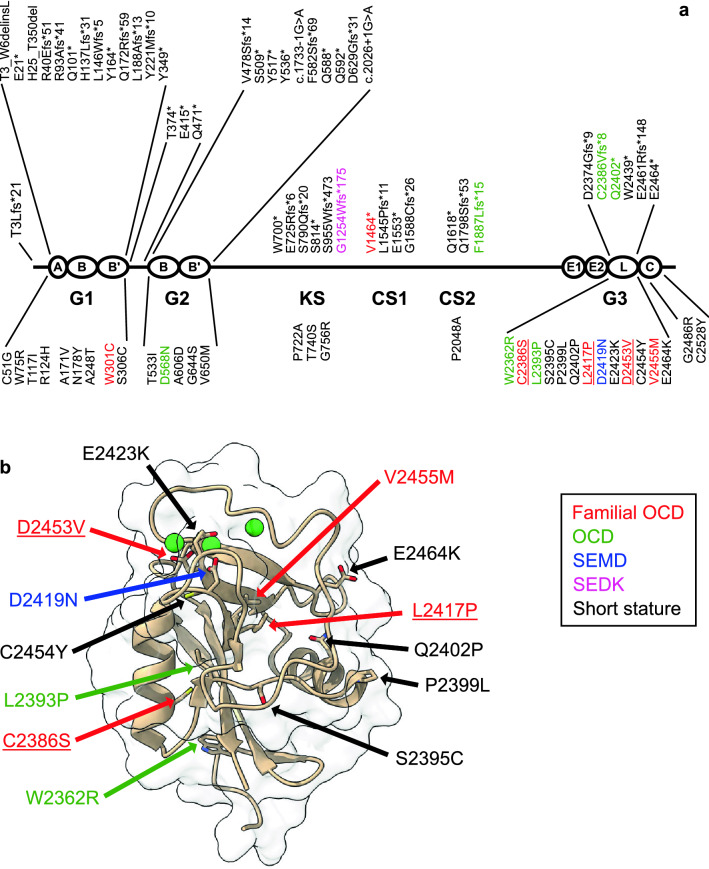
Hereditary *ACAN* variants segregating with skeletal disorders. (**a**) *ACAN* variants (published until October 2021) are shown schematically on the domain structure of the aggrecan core protein. Missense variants are shown below the core protein, all other types of variants above. Variants linked to familial OCD are shown in red, SEMD in blue and SEDK in pink. Variants where OCD has been reported in only one family member are shown in green. The novel fOCD variants reported in this study are underlined. (**b**) Locations of amino acid residues mutated in missense *ACAN* variants shown on the structure of the G3 domain C-type lectin repeat. Green spheres represent coordinated calcium ions. *ACAN* variant colouring is the same as in (**a**).

The novel fOCD-linked missense *ACAN* variants all locate in the C-type lectin repeat of the aggrecan C-terminal globular G3 domain (Fig. 4a). They affect the same aggrecan domain as the previously described fOCD-linked variant p.V2455M, and are all dominantly inherited.

When visualized on the three-dimensional structure of the aggrecan C-type lectin repeat (Protein Data Bank 1TDQ)^22^, the novel fOCD-linked variants affect amino acid residues in the hydrophobic core of the protein or residues coordinating calcium ions critical for structure and function of ligand binding loops on the protein surface (Fig. 4b). Additional, previously reported, missense variants where the proband shows OCD (p.W2362R, p.L2393P), also affect hydrophobic core residues^8,15,17^. The SEMD-linked variant (p.D2419N) affects one of the calcium-coordinating residues, replacing Asp with Asn, and thus forming a novel N-glycosylation site. Variants associated with short stature, but not OCD, appear more frequently at polar or charged amino acid residues exposed on the protein surface.

### Secretion of the fOCD variant aggrecan proteins is decreased

**Figure 5:**
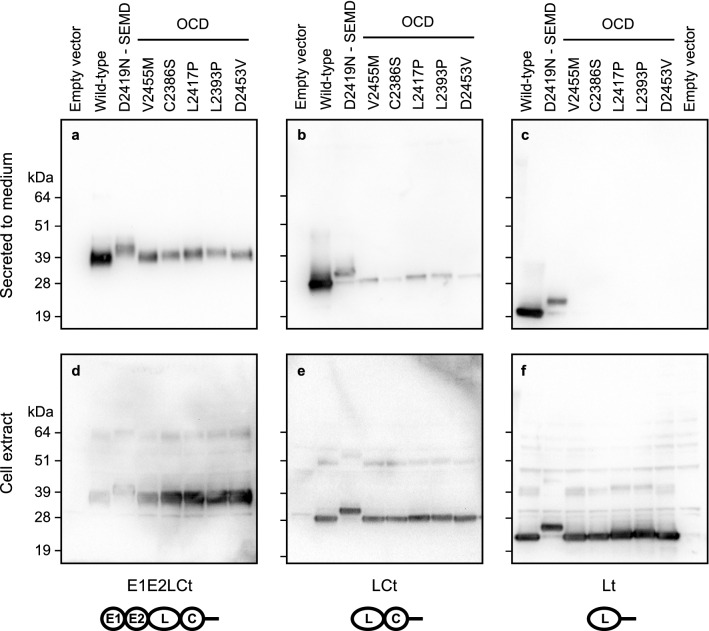
Secretion of recombinant aggrecan variant G3 domain protein. Expression plasmids for aggrecan SEMD and fOCD gene variants were transfected into human 293-c18 cells. Recombinant protein was recovered from culture medium (**a**–**c**) and cell lysates (**d**–**f**), and analyzed by immunoblotting. Each gene variant was tested in three different constructs corresponding to aggrecan G3 domain splice-forms; full-length E1E2LCt (**a**,**d**), C-type lectin and complement regulatory/sushi repeat LCt (**b**,**e**) and C-type lectin alone Lt (**c**,**f**). The domain arrangements of the G3 splice-forms tested are shown schematically at the bottom of the figure. As shown in (**d**,**e)**, all gene variants in all splice-forms tested were produced. Secretion of variant full-length G3 domain E1E2LCt was somewhat decreased compared to wild-type (**a**), while secretion of fOCD variants as LCt protein was strongly decreased (**b**). No secretion of any fOCD variant Lt protein, i.e. the C-type lectin alone, was observed (**c**). Note that the SEMD variant p.D2419N protein was secreted in all splice-forms, although at lower levels than wild-type protein (**a**–**c**).

To investigate effects on aggrecan function, we produced recombinant G3 domain proteins with the novel aggrecan variants, along with previously published C-type lectin repeat variants and the corresponding wild-type protein for comparison. To cover the range of domain sizes resulting from alternative splicing, we expressed the full domain (E1E2LCt, where E stands for EGF repeat, L for C-type Lectin, C for Complement regulatory protein-like/sushi repeat, and t for a short C-terminal tail sequence), the most common (LCt) and the shortest (Lt) splice variants in human 293-c18 cells. All variants were expressed (Fig. 5d–f), but as shown in Fig. 5a–c, secretion of the different fOCD variants to the culture medium was only efficient when the C-type lectin repeat was stabilized by flanking repeats (E1E2LCt). Interestingly, the p.L2393P variant protein behaved like the fOCD variants, even though OCD was only described in the proband for this variant. In contrast, the SEMD (p.D2419N) variant proteins were secreted even as the C-type lectin repeat alone (Fig. 5c). The slower mobility of the p.D2419N variant proteins result from the attachment of a carbohydrate chain to the novel N-linked glycosylation site formed by the mutation.

**Figure 6:**
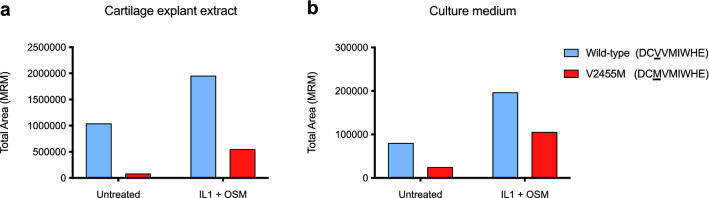
Decreased levels of fOCD variant aggrecan protein in patient cartilage. Cartilage tissue explants from a patient heterozygous for the fOCD-linked p.V2455M *ACAN* variant were obtained at arthroplasty. Some explants were treated with interleukin-1 and oncostatin M to stimulate protein production. After explant culture, the tissue explants were homogenized and extracted, and conditioned medium was collected. After GluC digestion, wild type and p.V2455M variant-specific peptides were quantified by mass spectrometry (MRM LC-MSMS) in the explant extracts (**a**) and medium (**b**). Note the decreased levels of p.V2455M variant compared to wild-type aggrecan proteoglycan in explant tissue (**a**) as well as in the culture medium (**b**).

We next wanted to clarify if these effects on fOCD-linked variant recombinant G3 domain secretion could be biologically relevant, i.e. if the variants also could affect secretion of the full-length aggrecan proteoglycan with the additional globular (G1, G2) interglobular and glycosaminoglycan-carrying regions present in addition to the G3 domain. In a pilot experiment, we cultured cartilage explants from an fOCD patient heterozygous for the p.V2455M variant, obtained at arthroplasty for advanced osteoarthritis. Using mass spectrometry (MRM LC-MSMS) directed at variant specific peptides, we quantified the wild-type and the variant proteins in the tissue explants (Fig. 6a) and in the conditioned culture medium (Fig. 6b). Should the presence of the entire proteoglycan counteract the effects of the variant amino acid residue in the G3 domain, we would expect to detect equal amounts of wild-type and fOCD variant proteins, since the tissue donor was heterozygous for the fOCD variant gene. The variant protein was, however, only present at low levels, both in the tissue explant (Fig. 6a) and the culture medium (Fig. 6b). Some explants were also treated with interleukin-1 and oncostatin M to stimulate cartilage protein release^23^. This resulted in increased levels of both wild-type and variant aggrecan, and while the ratio of variant aggrecan relative to wild-type were increased, variant aggrecan levels remained considerably lower than wild-type aggrecan.

### The novel ACAN variants affect aggrecan protein interactions

**Figure 7:**
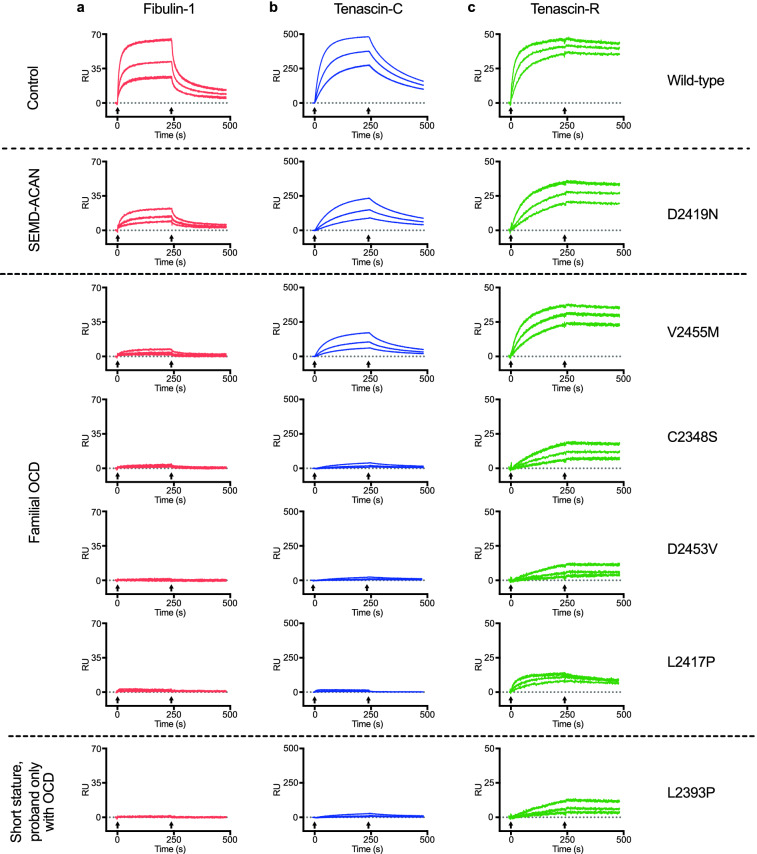
Impaired interactions of fOCD variant aggrecan G3 domains. Biacore surface plasmon resonance sensorgrams showing binding and dissociation of recombinant aggrecan G3 domains to immobilized cartilage ligands fibulin-1 (**a**) and tenascin-C (**b**) and the central nervous system ligand tenascin-R (**c**). Recombinant aggrecan G3 variants (E1E2LCt splice-form) injected at 50, 100 or 200 nM over the immobilised ligand surfaces. Duplicate injections of each concentration are shown in the graphs, with the start and end of injections marked by arrows under each curve. Note pronounced loss of binding of fOCD variants to fibulin-1 and tenascin-C (**a**,**b**). The *SEMD-ACAN* variant p.D2419N shows less affected binding, whereas as an *ACAN* variant linked to idiopathic short stature with OCD in the proband (p.L2393P) shows binding similar to the fOCD variants. Interaction with the central nervous system ligand tenascin-R (**c**) is less affected than the cartilage ligands, with residual binding of all aggrecan variants.

The aggrecan C-type lectin repeat has strong affinities for several extracellular matrix ligands^24–27^. In earlier studies, decreased ligand binding affinities of the p.V2455M fOCD and p.D2419N SEMD variant C-type lectins were observed^7,9^. We now purified the recombinantly produced aggrecan G3 domains (E1E2LCt splice form) described above, and performed BIAcore surface plasmon resonance binding assays with the known cartilage ligands tenascin-C and fibulin-1, as well as the central nervous system-specific ligand tenascin-R (Fig. 7). As shown earlier, the SEMD and p.V2455M fOCD variant proteins showed lower binding to tenascin-C and fibulin-1. Interestingly, the novel fOCD variants and the OCD variant p.L2393P all showed even further reduced binding. Interaction with the brain ligand tenascin-R was affected to a lesser degree, with retained binding of all the variants tested.

## Discussion

The aggrecan proteoglycan is an extracellular matrix component fundamental for the structure and function of all cartilages, including both in the growth plate during endochondral ossification and in adult articular cartilage. Disrupted aggrecan expression or function can thus cause developmental as well as adult phenotypes, or a combination of these. Growth plate phenotypes are evident in different hereditary disorders due to *ACAN* gene variants, with a phenotypic spectrum ranging from mild idiopathic short stature to severe chondrodysplasia. Variants affecting articular cartilage function appear less common, but variants linked to hereditary OCD or early onset osteoarthritis (in addition to short stature) may reflect different etiologies than variants affecting skeletal growth alone.

Most *ACAN* variants linked to growth plate phenotypes are autosomal dominant inherited nonsense or frameshift mutations and may affect any domain of aggrecan. These variants cause premature termination of translation and likely nonsense-mediated decay of the transcript, and result in decreased levels of aggrecan proteoglycan. Naturally, the decreased aggrecan levels caused by these variants may also affect articular cartilage function.

An interesting exception is the recessively inherited SEMD-linked missense mutation p.D2419N, which causes severe chondrodysplasia when present on both alleles^7^. This may reflect the combined reduction in aggrecan level resulting from both alleles being mutated, since individuals heterozygous for the variant had mild, proportionate short stature, with height around 150 cm^7^. Indeed, another case of SEMD was shown to result from compound heterozygosity for two different *ACAN* missense variants in the extended chondroitin sulfate chain attachment region^28^. The considerably milder phenotype of the compound heterozygous patient, however, suggests that effects on the G3 domain is an important component in the p.D2419N SEMD pathogenesis.

In contrast to nonsense and frameshift variants, the severity of missense mutations is difficult to predict and these are often classified as variants of unknown significance (VUS). In *ACAN*, missense mutations appear concentrated to the exons coding for folded, globular domains of the aggrecan core protein (Table 3). This may simply reflect that a missense mutation in any of the glycosaminoglycan attachment sites of the non-folded parts of the core protein would only result in the loss of one of approximately a hundred glycosaminoglycan chains. This marginal effect on aggrecan function would likely not result in an overt phenotype.

**Table 3:** Distribution of variants in *ACAN* regions (updated December 2021).

*ACAN* region	Total identified variants^a^	Missense variants	Total OCD variants^b^	Familial OCD variants^c^	Fraction missense of all variants (%)^d^	Fraction OCD of all variants (%)^d^	Fraction fOCD of all variants (%)^d^
Non-globular^e^	24	4	2	1	16.7	8.3	4.2
G1 domain	21	10	1	1	47.6	4.8	4.8
G2 domain	15	5	1	0	33.3	6.7	0
G3 domain^f^	21	15	8	4	71.4	38.1	19.0
Total	81	34	12	6	42.0	14.8	7.4

Note over-representation of familial and sporadic OCD variants in G3 domain.

^a^Including only variants with evidence for heredity and segregating with disease (see Supplemental Table 1 for additional variants).

^b^*ACAN* variants where proband presents with OCD.

^c^*ACAN* variants linked to hereditary OCD, i.e. found in proband and other variant-carrying family members.

^d^Percent of total variants in the region.

^e^Including intron 1, signal peptide, IGD (interglobular domain) and the KS and CS attachment regions.

^f^Of the G3 variants, 19 (including all fOCD and OCD variants) locate in the C-type lectin repeat.

The aggrecan globular domains, on the other hand, mediate interactions important for cartilage extracellular matrix assembly and organization. The fOCD-linked missense variants described in this paper obliterate interactions with known cartilage extracellular matrix ligands, as was shown earlier for the fOCD-linked p.V2455M variant^9^. Interestingly, the residues mutated in the fOCD-linked variants or the p.L2393P and p.W2263R variants (where the probands showed OCD) are not exposed on the surface of the folded protein (Fig. 4b). Indeed, neither of the mutated residues are part of the intermolecular binding surface of the aggrecan C-type lectin and tenascin-R^22^, suggesting that the loss of ligand interactions result from misfolding of the domain. Supporting this, we found that the secretion of fOCD variant proteins is decreased, both in cell culture models and in fOCD patient cartilage. This may be related to ER-stress and an unfolded protein response, as suggested by experiments on chondrocytes differentiated from mesenchymal stromal cells or induced pluripotent stem cells derived from a p.V2455M fOCD patient^29^. It seems reasonable to assume that the reduced aggrecan production contributes to the short stature due to effects in the growth plate. The impaired extracellular matrix interactions of fOCD variant aggrecan proteoglycans may contribute to poorer function or stability of cartilage tissue by affecting the molecular organization of the extracellular matrix. In addition, variant aggrecan proteolytic fragments lacking the G1 domain, and thus not anchored to hyaluronan, may be lost from the articular cartilage by diffusion to the joint space due to their failed G3 domain interactions, further decreasing aggrecan content of the tissue.

The novel *ACAN* variants described in this study were identified from families with hereditary OCD. Like the previously reported fOCD-linked variant p.V2455M^9^, the novel variants all segregate with OCD and idiopathic short stature, and at least for two of the variants with early onset osteoarthritis. In other studies, a further ten missense variants in the G3 domain have been linked to idiopathic short stature (Fig. 4)^8,13,15,17,18,30–33^. The probands of two of these families presented with OCD, but no heredity for OCD was reported for these families^8,15,17^. Thus, both fOCD and OCD variants are over-represented in the aggrecan G3 domain (Table 3). While the latter could simply reflect the normal prevalence of sporadic OCD, further OCD cases may yet appear in these families. Indeed, this is supported by the similarities in decreased secretion (Fig. 5) and impaired ligand interactions (Fig. 7) between the p.L2393P idiopathic short stature variant (with proband only showing OCD) and the fOCD variants. It is interesting to note that both these non-familial OCD missense variants (p.L2393P and p.W2362R) affect hydrophobic amino acid residues buried in the core of the folded domain, like the familial OCD variants p.C2386S, p.L2417P and p.V2455M (Fig. 4b). Based on this, we speculate that fOCD results from such variants disrupting the folding of the C-type lectin repeat in the aggrecan G3 domain, whereas variants affecting charged or polar residues exposed on the protein surface may be less detrimental.

Taken together, the fOCD and OCD linked to *ACAN* missense variants investigated in this study may constitute a subgroup of the SSOAOD classification. This may be considered as phenotypic variations of the same disorder or possibly reflect a different pathogenetic mechanism, although this would require further study. At present, the fOCD-linked missense variants, along with short stature and osteoarthritis-linked aggrecan variants have been classified together into one group (OMIM 165800, Short stature and advanced bone age, with or without early-onset osteoarthritis and/or osteochondritis dissecans). Previously, this group was termed “short stature, osteoarthritis and osteochondritis dissecans, SSOAOD”^34,35^. In the latest nosology of genetic skeletal disorders, this group is referred to as “short stature and advanced bone age, sometimes with osteochondritis dissecans”^36^. However, the inclusion of advanced bone age as a group criterion may have been premature, since later studies have shown that reduced or unchanged bone age frequently occur in idiopathic short stature associated with *ACAN* variants^9,11,15–17,20,21,26,28,30,31^.

To our knowledge, no other gene than *ACAN* has been linked to hereditary OCD. Interestingly, an additional fOCD missense *ACAN* variant has been reported affecting the aggrecan G1 globular domain^8,13^. This suggests that the aggrecan globular domains, and perhaps the G3 domain in particular, may be key to understanding the etiology of hereditary OCD and possibly also give insight into mechanisms behind sporadic OCD.

## Methods

### Ethical approval

All participants or their legal guardians gave informed consent for research and publication.

The study was first approved by the Regional Ethical Review Board at Umeå University, Sweden (Dnr 01-244 and 07-109M), and later by the Regional Ethical Review Board at Uppsala University, Sweden (Dnr 2018/189), and all research was performed in accordance with the relevant guidelines and regulations.

### Molecular genetic analysis

Genomic DNA from two index patients (family 1 and 2) were analyzed with an ABI Prism 3170 DNA sequencer at the Department of Clinical Genetics, Umeå University Hospital Umeå, Sweden. Bidirectional sequencing of exons and intron–exon boundaries of the *ACAN* gene (NM_00135.2, NM_013227.2) were performed as previously described^9^. The variants in *ACAN* were validated using Sanger sequencing on DNA from the index patient, and family members. A sample from a third index patient (family 3) was analyzed with NGS at Fulgent Diagnostics, Temple City, USA. Sanger sequencing was performed in family members for the variant identified in the index patient.

### Recombinant protein expression vector construction

An expression plasmid for recombinant wild-type aggrecan G3 domain fragments was constructed by inserting the cDNA into plasmid pCEP4-TAGzyme^37^, in frame and downstream of a BM40 signal peptide sequence followed by a hexa-histidine affinity tag.

Expression plasmids for aggrecan sequence variants were constructed through site-directed mutagenesis of the wild-type aggrecan expression plasmid, using the QuikChange mutagenesis system (Stratagene). The correct sequence of all constructs was verified by DNA sequencing of the entire inserts.

### Aggrecan variant recombinant protein secretion in cell culture

Human 293-c18 cells (ATCC, CRL-10852) in monolayer culture were transfected with plasmids expressing wild-type, SEMD-linked (p.D2419N) or different fOCD- or OCD-linked (p.V2455M, p.C2386S, p.L2393P, p.L2417P or pD2453V) aggrecan variants, using FuGene6 (Roche). After 48 h, the culture medium was collected and the cells harvested by trypsination, resuspended in PBS with soy bean trypsin inhibitor, washed by centrifugation and resuspended in PBS. The cell pellet was finally resuspended in 150 μl PBS with 2 mM MgCl_2_ and protease inhibitors (Complete w/o EDTA, Roche) and lysed with 20 U/μl Benzonase (Sigma) for 2 h at 37 °C. His-tagged proteins were enriched from conditioned media using NiNTA-coupled magnetic Dynabead precipitation (Invitrogen). The media and cell lysate proteins were separated by 4–20% SDS-PAGE gels (Novex), transferred onto PVDF membranes and probed with a rabbit antiserum directed to the C-type lectin repeat of the aggrecan G3 domain^26^, using chemiluminescent detection and documentation (Bio-Rad ChemiDoc MP). The images were processed by linear contrast enhancement, exported in TIF format, and the TIF files assembled to a figure and annotated using Affinity Design 1.7 software. The uncropped image files are shown in Supplemental Fig. S2.

### Aggrecan variant protein levels in FOCD patient cartilage

Knee cartilage from the tibial plateau was obtained at total knee replacement surgery, due to advanced osteoarthritis, from a 47 years old male patient heterozygous for the fOCD-linked p.V2455M aggrecan variant, with informed consent. Macroscopically normal cartilage was dissected and cultured as explants (in DMEM with 100ug/ml BSA, 50 ug/ml ascorbate, Penicillin/Streptomycin, Fungizone medium), with or without 10 ng/ml oncostatin M + 1 ng/ml Il-1 addition, as previously described^38^. Conditioned medium was collected and frozen at − 80 °C after 2 days in culture. The cartilage explant was snap-frozen in liquid nitrogen, pulverized and extracted with guanidine hydrochloride, as previously described^39^. After GluC digestion of the medium and cartilage extracts, the wild-type (DCVVMIWHE) and p.V2455M variant (DCMVMIWHE) peptides were quantified by multiple reaction monitoring (MRM) mass spectrometry using the top3 transitions (q1/q3) for each peptide (wild-type: (y7) = 594.76++/913.46+, (y6) = 594.76++/814.39+, (b3) = 594.76++/375.13+ and for V2455M: (y6) = 610.75++/814.39+, (y5) = 610.75++/715.32+ , (b3) = 610.75++/407.11+). The sample preparation and separation conditions were as previously described^40^.

### Recombinant protein production

Recombinant proteins were produced using the 293 Freestyle mammalian expression system (Invitrogen). Briefly, the expression plasmids were transiently transfected into human 293F cells using polyethylene imide “Max” (linear, MW 25 000, Polysciences Inc.)^41^, grown in suspension culture in Freestyle 293 medium in 8% CO_2_ atmosphere. The medium was harvested 7 days after transfection and recombinant proteins purified through nickel chelation affinity chromatography, followed by MonoQ ion exchange chromatography and gel filtration on a Superdex S200 Increase column using an ÄKTA chromatography system (Pharmacia, Uppsala, Sweden). The final gel filtration step was done with BIAcore running buffer (10 mM HEPES-HCl, 150 mM NaCl, 2 mM CaCl_2_, and 0.005% P20 [pH 7.5]) as eluent^9^.

### Protein interaction analysis

Interaction screening of the recombinant aggrecan variants were done by surface plasmon resonance, as previously described^9^. Briefly, recombinant fibulin-1, tenascin-C (fibronectin type III repeats 3–5) or tenascin-R (fibronectin type III repeats 3–5) were immobilized in CM5 flow cells to 811, 662 and 233 Resonance Units (RU), respectively. Recombinant aggrecan variant G3 domains (E1E2LCt), at 50, 100 or 200 nM in Biacore running buffer (see above), were flowed at 50 µl/min over these surfaces, and binding and dissociation followed in a BIAcore 2000 instrument (BIAcore, Uppsala, Sweden), and data analyzed with BIAevaluation 4.1 software.

## Supplementary Information

Supplementary Information.
