# Supplementary material for: Novel missense ACAN gene variants linked to familial osteochondritis dissecans cluster in the C-terminal globular domain of aggrecan
Source: Sci Rep. 2022 Mar 25;12:5215. doi: 10.1038/s41598-022-09211-y (PMC8956744; doi:10.1038/s41598-022-09211-y)
Supplement: Supplementary file 1 — Supplementary Information. [file 41598_2022_9211_MOESM1_ESM.pdf]

Supplementary information

Novel missense *ACAN* gene variants linked to familial osteochondritis dissecans cluster in the C-terminal globular domain of aggrecan

Eva-Lena Stattin[1#], Karin Lindblom[2], André Struglics[3], Patrik Önnerfjord[2], Jack Goldblatt[4], Abhijit Dixit[5], Ajoy Sarkar[5], Tabitha Randell[6], Mohnish Suri[5], Cathleen Raggio[7], Jessica Davis[7], Erin Carter[7] and Anders Aspberg[2]*

[1]Department of Immunology, Genetics and Pathology, Uppsala University, Uppsala, Sweden

[2]Rheumatology and Molecular Skeletal Biology, Department of Clinical Sciences Lund, Lund University, Sweden

[3]Orthopaedics, Department of Clinical Sciences Lund, Lund University, Lund, Sweden

[4]Genetic Services & Familial Cancer Program of Western Australia, King Edward Memorial Hospital for Women, Perth, WA, Perth, Australia

[5]Department of Clinical Genetics, Nottingham University Hospitals NHS Trust, Nottingham, United Kingdom

[6]Department of Paediatric Endocrinology, Nottingham University Hospitals NHS Trust, Nottingham, United Kingdom

[7]Kathryn O. and Alan C. Greenberg Center for Skeletal Dysplasias, Hospital for Special Surgery, New York, New York, USA

[#]Previously at the Department of Medical Biosciences, Medical and Clinical Genetics, Umeå University Hospital, Umeå, Sweden

*Address correspondence to:

Anders Aspberg, Rheumatology and Molecular Skeletal Biology, Department of Clinical Sciences Lund, Lund University, BMC-C12, 22184 Lund, Sweden.

E-mail address: anders.aspberg@med.lu.se

Supplemental table 1

Overview of skeletal effects of *ACAN* variants (December 2021)

cDNA level [1]	Protein level [1]	Affected domain	Phenotype [2]	Inheritance	Ref
Translocation t(10;15)	-	Intron 1	SS	AD	[1]
c.-7-2A>C*	-	Intron 1	SEDK	AD	[2]
c.6_13delCACTTTAC [4]	p.Thr3Leufs*21	Signal pept	SS	AD	[3]
c.6_17delinsTCT	p.Thr3_Trp6delinsLeu	Signal pept	SS	AD	[4]
c.61G>T	p.Glu21*	G1 A	SS, AdvBA, facial dysmorphism, mild skeletal effects	AD	[2, 5]
c.71_1051del	p.His25_Thr350del	G1 AB	SS, AdvBA, OA, Spine deformities	AD	[6]
c.116dupT	p.Arg40Glufs*51	G1 A	SS, facial dysmorphism, mild skeletal effects	AD	[7]
**c.151T>G** [5]	**p.Cys51Gly** [5]	G1 A	SS	AD	[8]
c.217delA	p.Ile73Serfs*12	G1 A	SS	Not analyzed	[9]
**c.223T>C**	**p.Trp75Arg**	G1 A	SS, AdvBA, OA, IDD	AD	[5]
c.272delA	p.Arg93Alafs*41	G1 A	SS, AdvBA, IDD, mild midface hypoplasia, short thumbs	AD	[5, 10]
c.301C>T	p.Gln101*	G1 A	SS, DelBA, OA, IDD	AD	[6]
**c.350C>T**	**p.Thr117Ile**	G1 A	SS	AD	[11]
**c.371G>A**	**p.Arg124His**	G1 A	SS, DelBA, mild skeletal defects	AD	[2, 12]
c.410_418delinsTGGA	p.His137Leufs*31	G1 A	SS, AdvBA	AD	[6]
c.436delC	pLeu146Trpfs*5	G1 A	SS	AD	[4]
c.492C>G	p.Tyr164*	G1 B	SS, AdvBA, OA, IDD	AD	[5]
**c.512C>T**	**p.Ala171Val**	G1 B	SS, AdvBA	AD	[13]
c.515delA	p.Gln172Argfs*59	G1 B	SS	AD	[8]
**c.532A>T**	**p.Asn178Tyr**	G1 B	SS, AdvBA	AD	[5, 14]
c.560dupA	p.Leu188Alafs*13	G1 B	SS, DelBA	AD	[11]
c.631_632insA	p.Tyr211* [3]	G1 B	SS, AdvBA	Not analyzed	[11]
c.661delT	p.Tyr221Metfs*10	G1 B	SS	AD	[3]
**c.742G>A**	**p.Ala248Thr**	G1 B	SS, mild skeletal defects	AD	[2]
**c.903G>C**	**p.Trp301Cys**	G1 B’	FOCD, SS, facial dysmorphism, mild skeletal effects, OA, IDD	AD	[2, 5, 12]

**c.916A>T**	**p.Ser306Cys**	G1 B’	SS, AdvBA, IDD	AD	[5]
**c.973G>A**	**p.Val325Met**	G1 B’	SS, craniofacial dysmorphism, kyphosis, trident hands	Not analyzed (compound heterozygous for c.2902T>C)	[15]
**c.1046A>G**	**p.Tyr349Cys**	G1 B’	SS only	Not analyzed	[16]
c.1047_1048delinsAC	p.Tyr349*	G1 B’	SS	AD	[5]
c.1117_1120delCAGA	p.Thr374*	IGD	SS	AD	[3]
c.1180C>T	p.Arg394*	IGD	FOCD, SS	Compound heterozygous for NPR2 mutation	[8]
c.1243G>T [3]	p.Glu415*	IGD	SS, OA, IDD	AD	[5]
c.1411C>T	p.Gln471*	IGD	SS	AD	[11, 17]
c.1425delA	p.Val478Serfs*14	G2 B	SS, OA	AD	[5]
c.1467C>G	p.Tyr489*	G2 B	SS, AdvBA	de novo	[11]
**c.1505G>C**	**p.Arg502Pro**	G2 B	SS only	Not analyzed	[16]
c.1526C>A	p.Ser509*	G2 B	SS, OA	AD	[5]
c.1531-1532delGA	p.Glu511Alafs*10	G2 B	SS	Not analyzed	[18]
c.1551C>G	p.Tyr517*	G2 B	SS	AD	[9]
**c.1598C>T**	**p.Thr533Ile**	G2 B	SEDK	AD	[2]
c.1608C>A	p.Tyr536*	G2 B	SS, AdvBA, mild osteochondral defects, OA, mild midface hypoplasia, broad great toes	AD	[2, 12, 19]
**c.1702G>A**	**p.Asp568Asn**	G2 B	OCD, SS	AD	[8]
c.1733-1G>A		G2 B	SS	AD	[17]
c.1744delT	p.Phe582Serfs*69	G2 B’	SS, IVD	AD	[20]
c.1762C>T	p.Gln588*	G2 B’	SS	AD	[17]
c.1774C>T	p.Gln592*	G2 B’	SS	AD	[8]
**c.1817C>A**	**p.Ala606Asp**	G2 B’	SS	AD	[17]
c.1861A>T	p.Lys621*	G2 B’	SS, AdvBA	Not analyzed	[11]
c.1880_1883dupTGGC	p.Asp629Glyfs*31	G2 B’	SS, AdvBA	AD	[11]
**c.1930G>A**	**p.Gly644Ser**	G2 B’	SS, mild skeletal defects	AD	[2, 12]
**c.1948G>A**	**p.Val650Met**	G2 B’	SS, DelBA, mild skeletal defects	AD	[2, 12]
**c.1979C>T**	**p.Thr660Met**	G2 B’	SS only	Not analyzed	[16]
c.2026+1G>A		G2 B’	SS, mild midface hypoplasia, brachydactyly	AD	[5, 10]

c.2099G>A	p.Trp700*	KS	SS, AdvBA, Spine deformities	AD	[6]
**c.2164C>G**	**p.Pro722Ala**	KS	SS	AD	[21]
c.2173delG	p.Glu725Argfs*6	KS	SS, AdvBA	AD	[11]
**c.2218A>T**	**p.Thr740Ser**	KS	SS, facial dysmorphism, mild skeletal effects	AD	[2, 12]
**c.2266G>C**	**p.Gly756Arg**	KS	SS	AD	[17]
c.2367delC	p.Ser790Glnfs*20	KS	SS, vertebral column,	AD	[7]
c.2369C>G	p.Ser790*	KS	SS, facial dysmorphism, mild skeletal effects	De novo	[2, 12]
c.2441C>G	p.Ser814*	KS	SS, early onset OA, vertebral column	AD	[22]
c.2535_2536insTTCA	p.Pro846Phefs*9	KS	SS only	Not analyzed	[16]
c.2863_2864delAG	p.Ser955Trpfs*473	GAG, CS-1	SS	AD	[4]
c.2902T>C	p.Ser968Pro	GAG, CS-1	SS, craniofacial dysmorphism, kyphosis, trident hands	Not analyzed (compound heterozygous for c.973G>A)	[15]
c.3758dupC	p.Gly1254Trpfs*175	GAG, CS-1	SEDK	AD	[23]
**c.4138G>T**	**p.Val1380Phe**	GAG, CS-1	SEMD (mild)	Not analyzed (compound heterozygour for c.5061T>A)	[24]
**c.4259A>G**	**p.Glu1420Gly**	GAG, CS-1	SS only	Not analyzed	[16]
c.4390delG	p.Val1464*	GAG, CS-1	fOCD, SS, craniofacial dysmorphism, brachydactyly	AD	[25]
c.4634delT	p.Leu1545Profs*11	GAG, CS-1	SS, IDD	AD	[26]
c.4657G>T	p.Glu1553*	GAG, CS-1	SS, OA, IDD	AD	[5]
c.4762_4765delGGGT	p.Gly1588Cysfs*26	GAG, CS-1	SS, AdvBA, mild midface hypoplasia, broad great toes	AD	[19]
c.4852C>T	p.Gln1618*	GAG, CS-2	SS, DelBA, OA	AD	[27]
**c.5061T>A**	**p.Ser1687Arg**	GAG, CS-2	SEMD (mild)	Not analyzed (compound heterozygous for c.4138G>T	[24]
c.5391delG	p.Gln1798Serfs*53 [3]	GAG, CS-2	SS, AdvBA,	AD	[5, 28]

			mild midface hypoplasia		
c.5443delC	p.Leu1815Trpfs*36	GAG, CS-2	SS, DelBA	Not analyzed	[11]
c.5579delC	p.Gly1861Glufs*41	GAG, CS-2	SS, AdvBA	Not analyzed	[11]
c.5597C>A	p.Ser1866*	GAG, CS-2	SS	de novo	[8]
c.5658delG	p.Phe1887Leufs*15	GAG, CS-2	OCD, SS	AD	[29]
**c.6142C>G**	**p.Pro2048Ala**	GAG, CS-2	SS, mild skeletal defects	AD	[2, 12]
c.6404delC	p.Ala2135Aspfs	GAG, CS-2	Mild SS, AdvBA	de novo	[27]
**c.6530T>C**	**p.Val2177Ala**	GAG, CS-2	SS only, polymorphism	Not analyzed	[16]
c.6861delC	p.Cys2288Valfs*28	G3, EGF1	SS, DelBA	Not analyzed	[11]
c.7121dup (c.7007dup)	p.Asp2374Glyfs*9 (p.Asp2337Glyfs*9)	G3, CLD	SS, AdvBA	AD	[30]
**c.7084T>C** (c.6970T>C)	**p.Trp2362Arg** (p.Trp2324Arg)	G3, CLD	OCD, SS, facial dysmorphism, mild skeletal effects	AD	[31]
c.7085G>A (c.6971G>A	p.Trp2362* (p.Trp2324*)	G3, CLD	SS	Not analyzed	[18]
c.7155delG (c.7041delG)	p.Cys2386Valfs*8 (p.Cys2348Valfs*8)	G3, CLD	OCD, SS, DelBA, OA	AD	[6]
**c.7156T>A** (c.7042T>A)	**p.Cys2386Ser** (p.Cys2348Ser)	G3, CLD	FOCD, SS, OA	AD	This study
**c.7178T>C** (c.7064T>C)	**p.Leu2393Pro** (p.Leu2355Pro)	G3, CLD	OCD, SS, AdvBA, OA, short thumbs	AD	[5, 10]
**c.7183A>T** (c.7069A>T)	**p.Ser2395Cys** (p.Ser2357Cys)	G3, CLD	SS, DelBA	AD	[6]
**c.7196C>T**	**p.Pro2399Leu**	G3, CLD	SS	AD	[11]
c.7204C>T (c.7090C>T)	p.Gln2402* (p.Gln2364*)	G3, CLD	OCD, SS, AdvBA, OA, mild midface hypoplasia, joint problems, broad great toes	AD	[19]
**c.7205A>C** (c.7091A>C) [3]	**p.Gln2402Pro** (p.Gln2364Pro)	G3, CLD	SS, mild skeletal defects	AD	[32]
c.7207-7209delGAG (c.7093_7095delGAG)	p.Glu2403del (p.Glu2365del)	G3, CLD	SS only	Not analyzed	[16]
**c.7250T>C** (c.7136T>C)	**p.Leu2417Pro** (p.Leu2379Pro)	G3, CLD	FOCD, SS, OA	AD	This study
**c.7255G>A** (c.7141G>A)	**p.Asp2419Asn** (p.Asp2381Asn)	G3, CLD	SEMD	AR	[33]
**c.7267G>A** (c.7153G>A)	**p.Glu2423Lys** (p.Glu2385Lys)	G3, CLD	SS, OA, IDD	AD	[5]
c.7317G>A (c.7203G>A)	p.Trp2439* (p.Trp2401*)	G3, CLD	SS, OA, IDD	AD	[5]
c.7336dupA (c.7222dupA)	p.Asn2446Lysfs*22 (p.Asn2407Lysfs*22)	G3, CLD	SS, AdvBA	Not analyzed	[34]
**c.7358A>T** (c.7244A>T)	**p.Asp2453Val** (p.Asp2415Val)	G3, CLD	FOCD, SS, OA	AD	This study

**c.7361G>A** (c.2416G>A)	**p.Cys2454Tyr** (p.Cys2416Tyr)	G3, CLD	SS	AD	[35]
**c. 7363G>A** (c.7249G>A)	**p.Val2455Met** (p.Val2417Met)	G3, CLD	FOCD, SS, OA, IDD	AD	[5, 36]
c.7381delG (c.7267delG) [3]	p.Glu2461Argfs*148 (p.Glu2423Argfs*148)	G3, CLD	SS, AdvBA, facial dysmorphism, mild skeletal effects	AD	[2]
**c.7390G>A** (c.7276G>A)	**p.Glu2464Lys** (p.Glu2426Lys)	G3, CLD	SS, facial dysmorphism, mild skeletal effects	AD	[2, 12]
c.7390G>T (c.7276G>T)	p.Glu2464* (p.Glu2426*)	G3, CLD	SS, facial dysmorphism, mild skeletal effects	AD	[2, 5]
**c.7456G>A** (c.7342G>A)	**p.Gly2486Arg** (p.Gly2448Arg)	G3, Sushi	SS, facial dysmorphism, mild skeletal effects	AD	[2]
**c.7583G>A** (c.7469G>A)	**p.Cys2528Tyr** (p.Cys2490Tyr)	G3, Sushi	SS	AD	[17]

[1] Sequence numbering is based on the full length ACAN variant 3 (NM_001369268.1). This sequence is identical to ACAN variant 2 (NM_013227.3) until nucleotide 6946, when the alternatively spliced EGF2 repeat is inserted in variant 3. For entries after this point, numbering based on NM_013227.3 is given in parenthesis.

[2] SS, short stature
SS only, no craniofacial, skeletal or joint symptoms and parents of normal height.
AdvBA, advanced bone age vs chronological age
DelBA, delayed bone age vs chronological age
OA, osteoarthritis
OCD, osteochondritis dissecans (reported only for proband)
fOCD, familiar OCD linked to the ACAN variant
IDD, intervertebral disc disease

[3] Labeled differently in original publication

[4] Gray background indicates non-globular parts of the aggrecan core protein

[5] Bold typeface marks missense variants

Referenses for Supplemental Table 1

1. Crippa, M., et al., *A balanced reciprocal translocation t(10;15)(q22.3;q26.1) interrupting ACAN gene in a family with proportionate short stature.* J Endocrinol Invest, 2018. **41**(8): p. 929-936.
2. Sentchordi-Montane, L., et al., *Heterozygous aggrecan variants are associated with short stature and brachydactyly: Description of 16 probands and a review of the literature.* Clin Endocrinol (Oxf), 2018. **88**(6): p. 820-829.
3. Hu, X., et al., *Novel pathogenic ACAN variants in non-syndromic short stature patients.* Clin Chim Acta, 2017. **469**: p. 126-129.
4. Hu, X., et al., *Proband-only medical exome sequencing as a cost-effective first-tier genetic diagnostic test for patients without prior molecular tests and clinical diagnosis in a developing country: the China experience.* Genet Med, 2018. **20**(9): p. 1045-1053.
5. Gkourogianni, A., et al., *Clinical characterization of patients with autosomal dominant short*

stature due to aggrecan mutations. J Clin Endocrinol Metab, 2016: p. jc20163313.
6. Stavber, L., et al., *High frequency of pathogenic ACAN variants including an intragenic deletion in selected individuals with short stature.* Eur J Endocrinol, 2020. **182**(3): p. 243-253.
7. Wei, M., et al., *Identification of novel ACAN mutations in two Chinese families and genotype-phenotype correlation in patients with 74 pathogenic ACAN variations.* Mol Genet Genomic Med, 2021: p. e1823.
8. Hauer, N.N., et al., *Genetic screening confirms heterozygous mutations in ACAN as a major cause of idiopathic short stature.* Sci Rep, 2017. **7**(1): p. 12225.
9. Ahn, J., et al., *Next-generation sequencing-based mutational analysis of idiopathic short stature and isolated growth hormone deficiency in Korean pediatric patients.* Mol Cell Endocrinol, 2021: p. 111489.
10. Nilsson, O., et al., *Short stature, accelerated bone maturation, and early growth cessation due to heterozygous aggrecan mutations.* J Clin Endocrinol Metab, 2014. **99**(8): p. E1510-8.
11. Lin, L., et al., *A High Proportion of Novel ACAN Mutations and Their Prevalence in a Large Cohort of Chinese Short Stature Children.* J Clin Endocrinol Metab, 2021. **106**(7): p. e2711-e2719.
12. Sentchordi-Montané, L., et al., *High prevalence of variants in skeletal dysplasia associated genes in individuals with short stature and minor skeletal anomalies.* Eur J Endocrinol, 2021.
13. Kim, T.Y., et al., *Identification of a heterozygous ACAN mutation in a 15-year-old boy with short stature who presented with advanced bone age: a case report and review of the literature.* Ann Pediatr Endocrinol Metab, 2020. **25**(4): p. 272-276.
14. Freire, B.L., et al., *Multigene Sequencing Analysis of Children Born Small for Gestational Age With Isolated Short Stature.* J Clin Endocrinol Metab, 2019. **104**(6): p. 2023-2030.
15. Panigrahi, I., et al., *Phenotypic heterogeneity of kyphoscoliosis with vertebral and rib defects: a case series.* Clin Dysmorphol, 2019. **28**(3): p. 103-113.
16. Hattori, A., et al., *Next generation sequencing-based mutation screening of 86 patients with idiopathic short stature.* Endocr J, 2017. **64**(10): p. 947-954.
17. Liang, H., et al., *Growth-Promoting Therapies May Be Useful In Short Stature Patients With Nonspecific Skeletal Abnormalities Caused By Acan Heterozygous Mutations: Six Chinese Cases And Literature Review.* Endocr Pract, 2020. **26**(11): p. 1255-1268.
18. He, D., et al., *Molecular diagnosis for growth hormone deficiency in Chinese children and adolescents and evaluation of impact of rare genetic variants on treatment efficacy of growth hormone.* Clin Chim Acta, 2021. **524**: p. 1-10.
19. van der Steen, M., et al., *ACAN Gene Mutations in Short Children Born SGA and Response to Growth Hormone Treatment.* J Clin Endocrinol Metab, 2017. **102**(5): p. 1458-1467.
20. Dateki, S., et al., *Identification of a novel heterozygous mutation of the Aggrecan gene in a family with idiopathic short stature and multiple intervertebral disc herniation.* J Hum Genet, 2017. **62**(7): p. 717-721.
21. Wang, Y., et al., *Short stature with precocious puberty caused by aggrecan gene mutation: A case report.* Medicine (Baltimore), 2020. **99**(34): p. e21635.
22. Ruault, V., et al., *Clinical and Molecular Spectrum of Nonsyndromic Early-Onset Osteoarthritis.* Arthritis Rheumatol, 2020. **72**(10): p. 1689-1693.
23. Gleghorn, L., et al., *A mutation in the variable repeat region of the aggrecan gene (AGC1) causes a form of spondyloepiphyseal dysplasia associated with severe, premature osteoarthritis.* Am J Hum Genet, 2005. **77**(3): p. 484-90.
24. Fukuhara, Y., et al., *The second report on spondyloepimetaphyseal dysplasia, aggrecan type: a milder phenotype than originally reported.* Clin Dysmorphol, 2019. **28**(1): p. 26-29.
25. Mancioppi, V., et al., *Retrospective Diagnosis of a Novel ACAN Pathogenic Variant in a Family With Short Stature: A Case Report and Review of the Literature.* Front Genet, 2021. **12**: p. 708864.
26. Uchida, N., et al., *A novel mutation in the ACAN gene in a family with autosomal dominant short stature and intervertebral disc disease.* Hum Genome Var, 2020. **7**(1): p. 44.
27. Tatsi, C., et al., *Aggrecan Mutations in Nonfamilial Short Stature and Short Stature Without Accelerated Skeletal Maturation.* J Endocr Soc, 2017. **1**(8): p. 1006-1011.

28. Quintos, J.B., M.H. Guo, and A. Dauber, *Idiopathic short stature due to novel heterozygous mutation of the aggrecan gene.* J Pediatr Endocrinol Metab, 2015. **28**(7-8): p. 927-32.
29. Denis, A., et al., *Variable expressivity in a family with an aggrecanopathy.* Mol Genet Genomic Med, 2021: p. e1773.
30. Ye, X., et al., *Dual diagnosis of osteogenesis imperfecta (OI) and short stature and advanced bone age with or without early-onset osteoarthritis and/or osteochondritis dissecans (SSOAOD) reveals a cumulative effect on stature caused by mutations in COL1A1 and ACAN genes.* Eur J Med Genet, 2020. **63**(12): p. 104074.
31. Florio, A., et al., *A child with a novel ACAN missense variant mimicking a septic arthritis.* Ital J Pediatr, 2019. **45**(1): p. 148.
32. Xu, D., et al., *Novel aggrecan variant, p. Gln2364Pro, causes severe familial nonsyndromic adult short stature and poor growth hormone response in Chinese children.* BMC Med Genet, 2018. **19**(1): p. 79.
33. Tompson, S.W., et al., *A recessive skeletal dysplasia, SEMD aggrecan type, results from a missense mutation affecting the C-type lectin domain of aggrecan.* Am J Hum Genet, 2009. **84**(1): p. 72-9.
34. Yang, L., et al., *Pathogenic gene screening in 91 Chinese patients with short stature of unknown etiology with a targeted next-generation sequencing panel.* BMC Med Genet, 2018. **19**(1): p. 212.
35. Benonisdottir, S., et al., *Epigenetic and genetic components of height regulation.* Nat Commun, 2016. **7**: p. 13490.
36. Stattin, E.L., et al., *A missense mutation in the aggrecan C-type lectin domain disrupts extracellular matrix interactions and causes dominant familial osteochondritis dissecans.* Am J Hum Genet, 2010. **86**(2): p. 126-37.

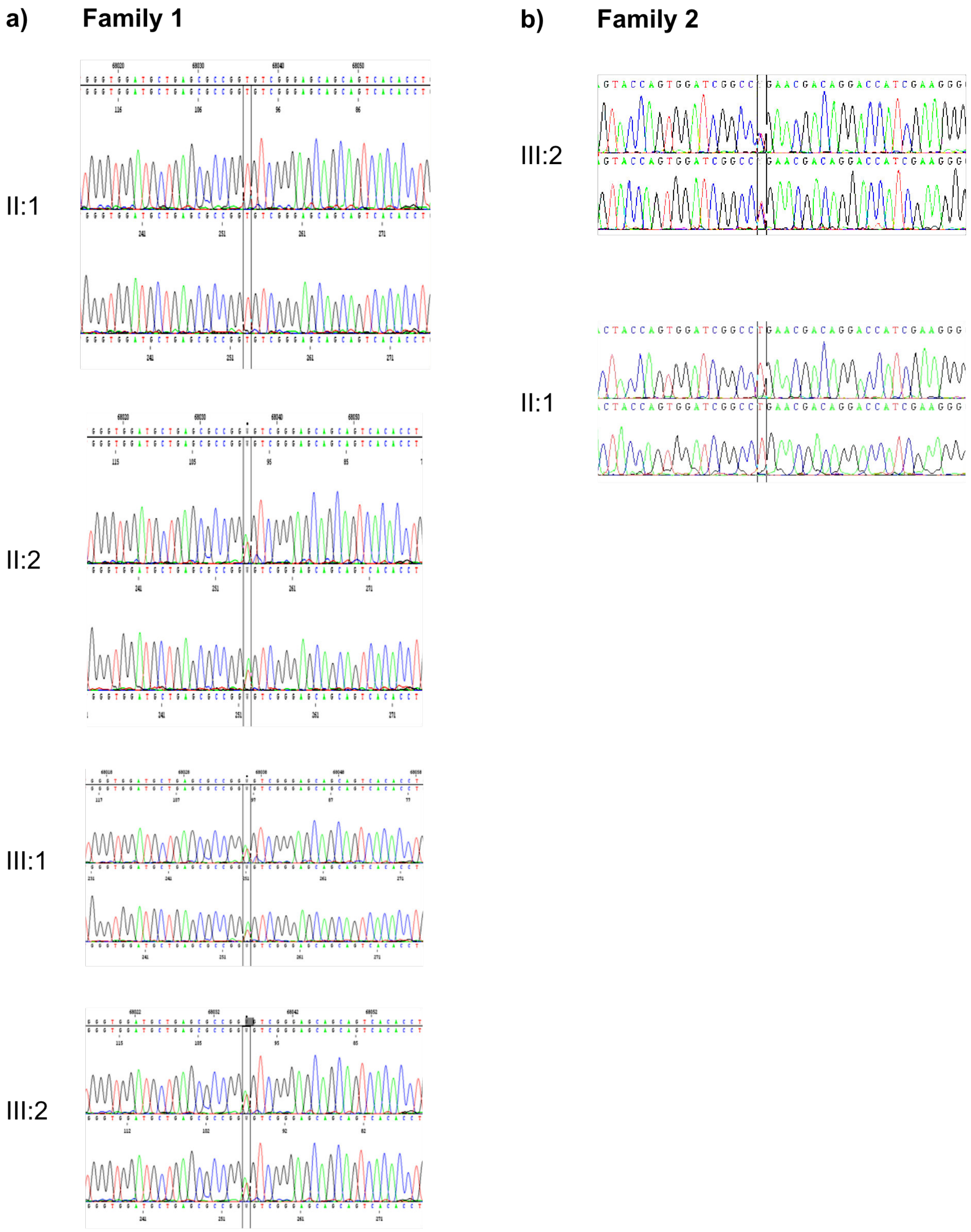

Supplemental figure 1. Genomic DNA sequencing

a) Sequence traces from Family 1 proband (III:1), sister (III:2), mother (II:2) and father (II:1).

b) Sequence traces from Family 2 proband (III:2) and father (II:1). The sibling (III:1) and mother (II:2) of the proband showed the same C>T variation as III:2.

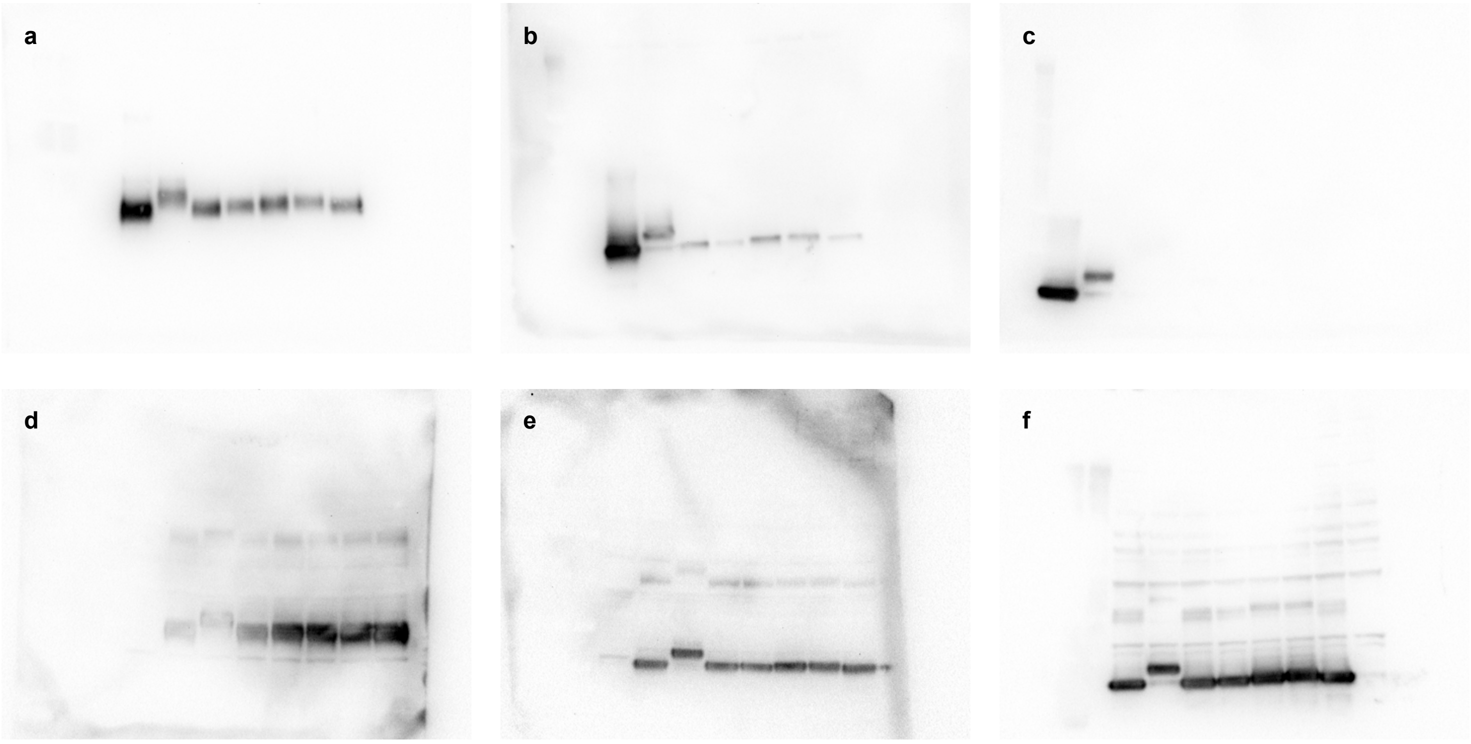

Figure S2. Un-cropped western blots.

Original digital images of western blots, shown cropped in Figure 5a-f.
